# Supplementary material for: Genome-Wide Association Mapping Identifies New Candidate Genes for Cold Stress and Chilling Acclimation at Seedling Stage in Rice (Oryza sativa L.)
Source: Int J Mol Sci. 2022 Oct 30;23(21):13208. doi: 10.3390/ijms232113208 (PMC9655271; doi:10.3390/ijms232113208)
Supplement: Supplementary file 1 [file ijms-23-13208-s001.zip › ijms-1951395-supplementary/supplement files/Supplemental Figures.pptx]

## Slide 1
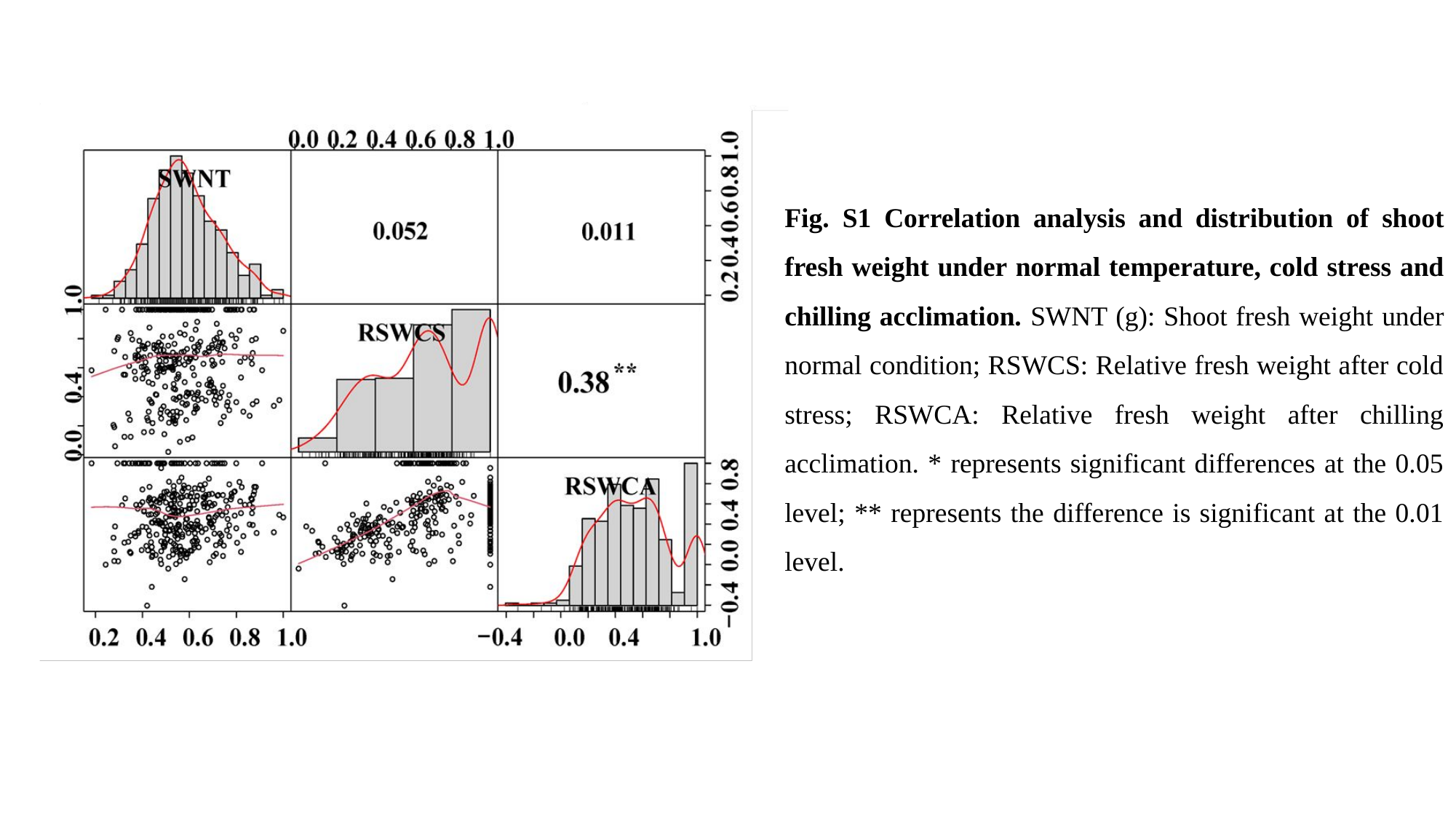

Fig. S1 Correlation analysis and distribution of shoot fresh weight under normal temperature, cold stress and chilling acclimation. SWNT (g): Shoot fresh weight under normal condition; RSWCS: Relative fresh weight after cold stress; RSWCA: Relative fresh weight after chilling acclimation. * represents significant differences at the 0.05 level; ** represents the difference is significant at the 0.01 level.
